# Supplementary material for: Seeking Help from Chatbots for Suicide Thoughts: Associations with Other Help Seeking Sources and Mental Health Symptoms
Source: medRxiv. 2026 Aug 14:2026.08.12.26360318. Preprint. [Version 1] doi: 10.64898/2026.08.12.26360318 (PMC13640871; doi:10.64898/2026.08.12.26360318)
Supplement: Supplementary file 1 [file media-1.pdf]

Appendix

Supplementary Table 1. Paired Comparison of Chatbot Help-Seeking Intentions with Other Sources

Source	Mean difference	Wilcoxon V	|*r*|	*p*
**Suicide Thoughts**				
Intimate partner	−2	22		<.001***
Friend	−2	143.5	0.51	<.001***
Parent	0	83	0.19	.161
Other family member	0	148.5	0.26	.051
Mental health professional	−3	37	0.53	<.001***
Helpline	0	109	0.31	.019*
General doctor	−1	150.5	0.49	<.001***
Religious leader	1	109.5	0.19	.156
**General Emotional Problems**				
Intimate partner	−2.5	31.5	0.73	<.001***
Friend	−2	206	0.52	<.001***
Parent	−0.31	186.5	0.16	.227
Other family member	0	227	0.21	.112
Mental health professional	−3	90.5	0.72	<.001***
Helpline	0	250.5	0.17	.194
General doctor	−2	161.5	0.59	<.001***
Religious leader	0	202	0.22	.102

Negative mean differences indicate lower intentions to seek help from chatbots than from the comparison source. Wilcoxon signed-rank tests were conducted to compare help-seeking intentions across topics: general emotional problems versus suicide thoughts. V refers to the Wilcoxon signed-rank test statistic. Effect sizes are reported as the absolute value of *r*. Following Cohen's thresholds for r, |r| values of .10 to < .30, 30 to <.50, and ^{3} .50 were interpreted as small, medium, and large effects, respectively.[28,29] *** $p < .001$, ** $p < .01$, * $p < .05$

Supplementary Table 2. Associations of Age, Sex, Race, and Ethnicity with Help-Seeking Intentions

	Suicide Thoughts		General Emotional Problems	
	Statistic τ/W	*p*	Statistic τ/W	*p*
**Chatbot**				
Age [a]	-0.26	.016*	-0.30	.004**
Sex [b]	419	.820	435	.631
Race [b]	446.5	.200	399	.049*
Ethnicity [b]	187	.699	140	.123
**Online**				
Age [a]	0.04	.874	-0.07	.486
Sex [b]	371.5	.449	363	.448
Race [b]	389	.844	463	.136
Ethnicity [b]	256	.095	196	.932
**Informal**				
Age [a]	0.03	.740	-0.06	.526
Sex [b]	276.5	.038	216.5	.003**
Race [b]	508	0.032*	607.5	<.0001***
Ethnicity [b]	243	.335	196	.937
**Formal**				
Age [a]	0.329	.832	0.01	.920
Sex [b]	350	.363	298	.083
Race [b]	500	.045*	486.5	.067
Ethnicity [b]	204	.937	169.5	.932

[a] Kendall's τ correlation. [b] Wilcoxon rank-sum tests. Wilcoxon rank-sum tests were conducted to examine between-group differences in help-seeking intentions across demographic groups. W denotes the Wilcoxon rank-sum test statistic. Sex was categorized as female or male; race as White or non-White; and ethnicity as Hispanic or non-Hispanic. *** $p < .001$, ** $p < .01$, * $p < .05$

Supplementary Table 3. Associations of Symptom Characteristics with Help-Seeking Intentions

		Suicide Thoughts		General Emotional Problems	
		τ/W	*p*	τ/W	*p*
**Chatbot**					
	PHQ [a]	0.01	.9112	-0.02	.865
	GAD [a]	0.33	.003**	0.31	.004*
	Lifetime suicide attempt [b]	361.5	.455	349.5	.397
	Past-month suicide ideation [b]	148	.112	162	.331
**Anonymous online**					
	PHQ [a]	0.09	.398	0.06	.585
	GAD [a]	0.06	.603	0.09	.392
	Lifetime suicide attempt [b]	329	.155	316.5	.172
	Past-month suicide ideation/behavior [b]	200.5	1	196.5	.942
**Informal**					
	PHQ [a]	-0.11	.234	-0.14	.154
	GAD [a]	0.06	.603	0.08	.395
	Lifetime suicide attempt [b]	370	.681	366	0.635
	Past-month suicide ideation/behavior [b]	265.5	.141	240.5	.365
**Formal**					
	PHQ [a]	0.01	.935	0.05	.571
	GAD [a]	0.06	.515	0.15	.133
	Lifetime suicide attempt [b]	358	.547	315.5	0.199
	Past-month suicide ideation/behavior [b]	213.5	.769	168	.476

[a] = Kendall tau [b] = Wilcoxon rank-sum test. Wilcoxon rank-sum tests were conducted to examine between-group differences in help-seeking intentions across CSSRS groups. W denotes the Wilcoxon rank-sum test statistic. *** $p < .001$, ** $p < .01$, * $p < .05$

Supplementary Figure 1. Correlations among Individual Help-seeking Sources

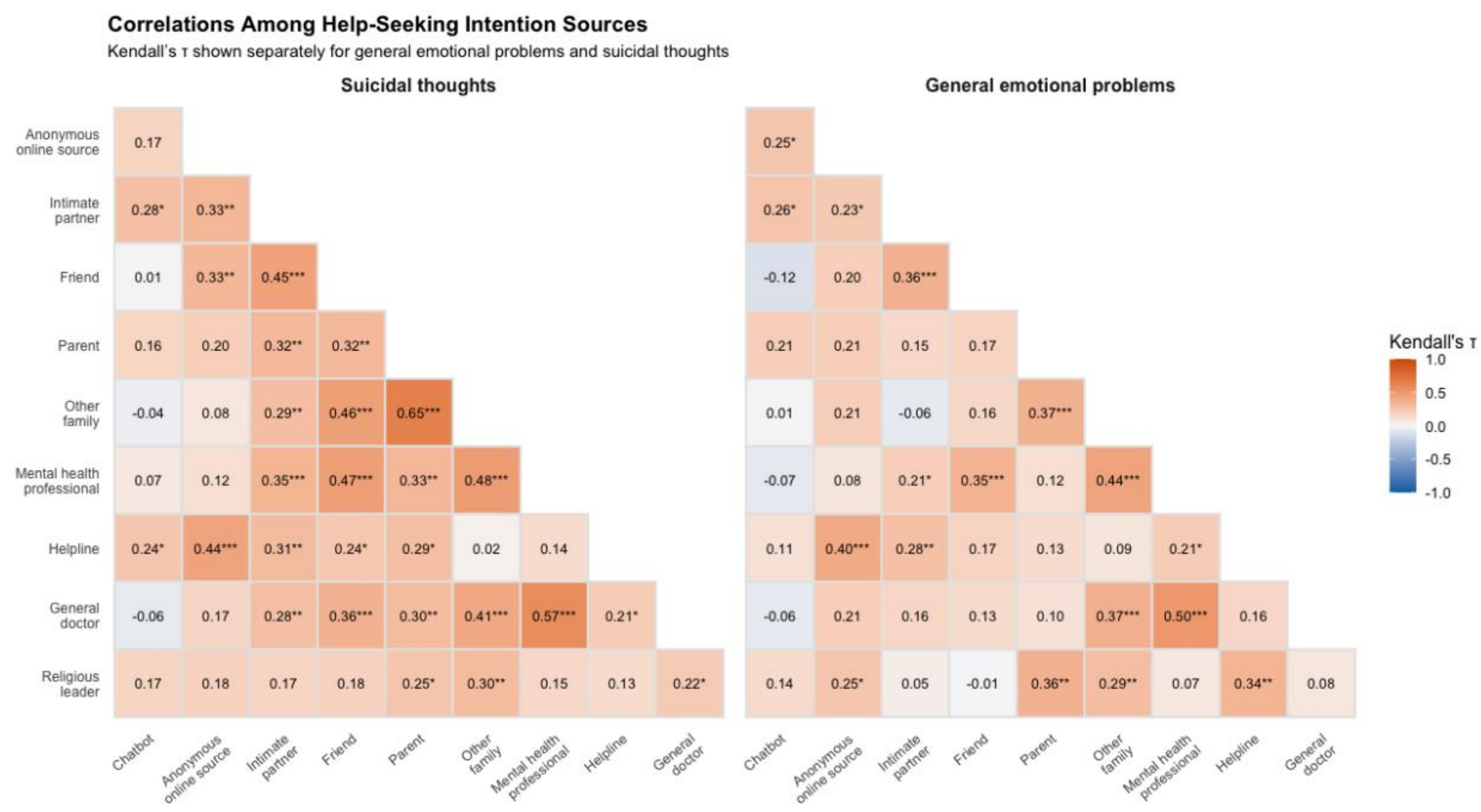

*** $p < .001$, ** $p < .01$, * $p < .05$
